# Common and distinct risk factors that influence more severe and distressing shortness of breath profiles in oncology outpatients

**DOI:** 10.1002/cam4.7013

**Published:** 2024-02-24

**Authors:** Joosun Shin, Marilyn Hammer, Mary E. Cooley, Bruce A. Cooper, Steven M. Paul, Frances Cartwright, Kord M. Kober, Yvette P. Conley, Jon D. Levine, Christine Miaskowski

**Affiliations:** ^1^ Dana‐Farber Cancer Institute Boston Massachusetts USA; ^2^ School of Nursing University of California San Francisco California USA; ^3^ Mount Sinai Medical Center New York New York USA; ^4^ School of Nursing University of Pittsburgh Pittsburgh Pennsylvania USA; ^5^ School of Medicine University of California San Francisco California USA

**Keywords:** cancer, chemotherapy, distress, dyspnea, shortness of breath

## Abstract

**Background:**

Shortness of breath occurs in 10%–70% of oncology patients. Very little is known about interindividual variability in its severity and distress and associated risk factors. Using latent profile analyses (LPAs), purpose was to identify subgroups of patients with distinct severity and distress profiles for shortness of breath as single symptom dimensions. In addition, a joint LPA was done using patients' severity AND distress ratings. For each of the three LPAs, differences among the shortness of breath classes in demographic, clinical, symptom, stress, and resilience characteristics were evaluated.

**Methods:**

Patients completed ratings of severity and distress from shortness of breath a total of six times over two cycles of chemotherapy. All of the other measures were completed at enrollment (i.e., prior to the second or third cycle of chemotherapy). Separate LPAs were done using ratings of severity and distress, as well as a joint analysis using severity AND distress ratings. Differences among the latent classes were evaluated using parametric and nonparametric tests.

**Results:**

For severity, two classes were identified (Slight to Moderate [91.6%] and Moderate to Severe [8.4%]). For distress, two classes were identified (A Little Bit to Somewhat [83.9%] and Somewhat to Quite a Bit [16.1%]). For the joint LPA, two classes were identified (Lower Severity and Distress [79.9%] and Higher Severity and Distress [20.1%]). While distinct risk factors were associated with each of the LPAs, across the three LPAs, the common risk factors associated with membership in the worse class included: a past or current history of smoking, poorer functional status, and higher comorbidity burden. In addition, these patients had a higher symptom burden and higher levels of cancer‐specific stress.

**Conclusions:**

Clinicians can use the information provided in this study to identify high‐risk patients and develop individualized interventions.

## 1 INTRODUCTION

A comprehensive evaluation of shortness of breath warrants assessment of its sensory‐perceptual experience, affective distress, and impact.^1^ The sensory‐perceptual experience is assessed using ratings of severity. In contrast, affective distress includes an evaluation of patients' perceptions of its unpleasantness and cognitive response to it.^1^ Based on our systematic review of shortness of breath in patients with cancer,^2^ of the 50 studies that assessed two symptom dimensions, only 5 reported on its severity and distress^3^, ^4^, ^5^, ^6^, ^7^ and only 1 measured distress using a numeric rating scale (NRS).^5^ Given the paucity of information on these two dimensions of shortness of breath in oncology patients, additional research is warranted not only on the dimensions themselves (e.g., changes over time); but on risk factors for higher levels of both severity and distress. Equally important, as shortness of breath rarely occurs in isolation,^8^ associations between each dimension and other common symptoms reported by oncology patients warrants evaluation.

Across 10 studies, significant risk factors for more severe shortness of breath included: older age,^9^, ^10^ lower socioeconomic status,^9^, ^10^, ^11^, ^12^ lower functional status,^11^, ^13^, ^14^, ^15^, ^16^ sedentary lifestyle,^12^ occurrence of cardiopulmonary comorbidities,^11^, ^12^, ^14^, ^17^ presence of advanced stage cancer,^9^, ^12^, ^16^, ^18^ occurrence of lung metastasis,^11^ a history of cancer treatment,^10^, ^12^ and the occurrence of anxiety and depression.^9^, ^10^, ^11^, ^16^, ^17^ However, these findings need to be interpreted with caution because the results were inconsistent^9^, ^10^, ^18^ and sample sizes were relatively small.^15^, ^17^ Of note, none of these studies evaluated for risk factors associated with higher levels of distress from shortness of breath.^2^

In terms of associations between shortness of breath and other common symptoms reported by oncology patients, only fourteen studies assessed relationships between its severity and anxiety,^3^, ^4^, ^11^, ^12^, ^14^, ^19^, ^20^, ^21^, ^22^, ^23^, ^24^ depression,^3^, ^4^, ^11^, ^12^, ^19^, ^20^, ^21^, ^22^, ^23^, ^25^ fatigue,^4^, ^19^, ^24^, ^25^, ^26^ pain,^4^, ^11^, ^13^, ^25^ and sleep disturbance.^4^, ^19^ Most of these analyses were limited to simple correlation coefficients. While informative, this analytic approach does not provide information on interindividual differences in the severity of common cancer‐related symptoms in patients with different severity and/or distress profiles from shortness of breath.

Stress is an additional risk factor that may contribute to worse severity and distress from shortness of breath. While no studies reported on associations between the severity and/or distress from shortness of breath and stress or resilience in oncology patients, in previous studies of pediatric patients with asthma^27^ and adult patients receiving supportive and palliative care,^28^ the severity of shortness of breath was positively correlated with psychological stress. In our recent study,^29^ patients with the highest occurrence rates for shortness of breath reported higher levels of global, cancer‐related, and cumulative life stress. Given that a cancer diagnosis and its associated treatments are extremely stressful experiences,^30^, ^31^ and albeit limited information on positive associations between the occurrence and severity of shortness of breath and stress, an examination of its associations with both severity and distress is warranted. Equally important, evidence suggests that resilience facilitates a positive cognitive appraisal of perceived stress.^32^ Therefore, in the current study, we hypothesize that lower levels of resilience will be associated with worse severity and distress profiles for shortness of breath.

In our previous work, that used the latent class analysis (LCA) to identify distinct shortness of breath profiles using ratings of symptom occurrence,^33^ four classes were identified (i.e., None, Decreasing, Increasing, and High). The current study extends our previous research and focuses on the characterization of interindividual differences in severity and distress from shortness of breath. Using latent profile analyses (LPAs), the purpose of this study was to identify subgroups of patients with distinct severity and distress profiles for shortness of breath as single symptom dimensions. Because previous research found that the symptoms that are the most severe are not the most distressing and vice versa,^34^, ^35^ a joint LPA was done using patients' severity AND distress ratings. For each of the three LPAs (i.e., only severity, only distress, and severity AND distress), differences among the shortness of breath classes in demographic, clinical, symptom, and stress characteristics were evaluated. Given that the goal of these analyses was to identify common and distinct risk factors for membership in the worse shortness of breath severity, distress, and joint classes, the findings from the three separate latent class analyses are compared and contrasted.

## 2 METHODS

### 2.1 Patients and settings

This study is part of a larger, longitudinal study of the symptom experience of oncology outpatients receiving chemotherapy. Eligible patients were ≥18 years of age; had a diagnosis of breast, gastrointestinal, gynecological, or lung cancer; had received chemotherapy within the preceding four weeks; were scheduled to receive at least two additional cycles of chemotherapy; were able to read, write, and understand English; and gave written informed consent. Patients were recruited from two Comprehensive Cancer Centers, one Veteran's Affairs hospital, and four community‐based oncology programs during their first or second cycle of chemotherapy. The major reason for refusal was being overwhelmed with their cancer treatment.

### 2.2 Study procedures

Study was approved by the Committee on Human Research at the University of California, San Francisco and Institutional Review Board at each of the study sites. Patients were approached by a research nurse in the infusion unit prior to their first or second cycle of chemotherapy. Of the 2234 patients approached, 1343 provided written informed consent to participate (60.1% response rate). Of these 1343 patients, 395 reported the occurrence of shortness of breath using the Memorial Symptom Assessment Scale (MSAS). Of these 395 patients, 381 rated its severity and 380 rated its distress a total of six times over two chemotherapy cycles (i.e., prior to chemotherapy administration (Assessments 1 and 4), approximately 1 week after chemotherapy administration (Assessments 2 and 5), and approximately 2 weeks after chemotherapy administration (Assessments 3 and 6)). Patients completed the other measures used in this analysis at enrollment (i.e., prior to the second or third cycle of chemotherapy).

### 2.3 Instruments

#### 2.3.1 Demographic and clinical measures

Patients completed a demographic questionnaire, Karnofsky Performance Status (KPS) scale,^36^ Self‐Administered Comorbidity Questionnaire (SCQ),^37^ Alcohol Use Disorders Identification Test (AUDIT),^38^ and smoking history questionnaire. Medical records were reviewed for disease and treatment information.

#### 2.3.2 Measure of the severity and distress of shortness of breath

The shortness of breath item from the MSAS was used to assess its severity (i.e., slight, moderate, severe, and very severe) and distress (i.e., not at all, a little bit, somewhat, quite a bit, and very much) using Likert scales at each of the six assessments. Validity and reliability of the MSAS are well established.^39^

#### 2.3.3 Symptom measures

The 20‐item Center for Epidemiological Studies‐Depression scale (CES‐D) evaluates the major symptoms in the clinical syndrome of depression. A total score can range from 0 to 60, with scores of ≥16 indicating the need for individuals to seek clinical evaluation for major depression. The CES‐D has well established validity and reliability.^40^, ^41^, ^42^ Its Cronbach's alpha was 0.89.

The 20 items on the Spielberger State–Trait Anxiety Inventory (STAI‐S and STAI‐T) were rated from 1 to 4.^43^ The STAI‐S measures a person's temporary anxiety response to a specific situation or how anxious or tense a person is “right now” in a specific situation. The STAI‐T measures a person's predisposition to anxiety as part of one's personality. Cutoff scores of ≥31.8 and ≥32.2 indicate a high level of trait and state anxiety, respectively. Cronbach's alphas for the STAI‐T and STAI‐S were 0.92 and 0.96, respectively.

The 18‐item Lee Fatigue Scale (LFS) was designed to assess physical fatigue and energy.^44^ Each item was rated on a 0–10 numeric rating scale (NRS). Total fatigue and energy scores were calculated as the mean of the 13 fatigue items and the 5 energy items, respectively. Higher scores indicate greater fatigue severity and higher levels of energy. Using separate LFS questionnaires, patients were asked to rate each item based on how they felt within 30 min of awakening (i.e., morning fatigue and morning energy) and prior to going to bed (i.e., evening fatigue and evening energy). The LFS has established cutoff scores for clinically meaningful levels of fatigue (i.e., ≥3.2 for morning fatigue and ≥5.6 for evening fatigue) and energy (i.e., ≤6.2 for morning energy and ≤3.5 for evening energy).^45^ Cronbach's alphas were 0.96 for morning and 0.93 for evening fatigue and 0.95 for morning and 0.93 for evening energy.

The 21‐item General Sleep Disturbance Scale (GSDS) was designed to assess the quality of sleep in the past week.^46^ Each item was rated on a 0 (never) to 7 (everyday) NRS. The GSDS total score is the sum of the 21 items that can range from 0 (no disturbance) to 147 (extreme sleep disturbance). Higher total scores indicate higher levels of sleep disturbance. A GSDS total score of ≥43 indicates a significant level of sleep disturbance.^45^ Cronbach's alpha for GSDS score was 0.83.

The 16‐item Attentional Function Index (AFI) assesses an individual's perceived effectiveness in performing daily activities that are supported by attention and working memory.^47^ A higher total mean score on a 0–10 NRS indicates better cognitive function.^47^ Total scores are grouped into categories of attentional function (i.e., <5 low function, 5.0–7.5 moderate function, and >7.5 high function).^48^ Cronbach's alpha for the total AFI score was 0.93.

The occurrence of pain was evaluated using the Brief Pain Inventory.^49^ Patients who responded yes to the question about having pain were asked to indicate if their pain was or was not related to their cancer treatment. Patients were categorized into one of four groups (i.e., no pain, only noncancer pain, only cancer pain, both cancer, and noncancer pain). Patients rated the intensity of their worst pain using 0 (none) to 10 (excruciating) NRS. In addition, they provided information on pain's level of interference with function.

#### 2.3.4 Stress and resilience measures

The 14‐item Perceived Stress Scale (PSS) was used as a measure of global perceived stress according to the degree that life circumstances are appraised as stressful over the course of the previous week.^50^ Each item was rated on a 0–4 Likert scale (i.e., 0 = never, 1 = almost never, 2 = sometimes, 3 = fairly often, and 4 = very often). Total PSS scores can range from 0 to 56. Its Cronbach's alpha was 0.89.

The 22‐item Impact of Event Scale‐Revised (IES‐R) was used to measure cancer‐related distress.^51^, ^52^ Patients rated each item based on how distressing each potential difficulty was for them during the past week “with respect to their cancer and its treatment.” Each item was rated on a 0 (not at all) to 4 (extremely) Likert scale. Three subscales evaluate levels of intrusion, avoidance, and hyperarousal perceived by the patient. The total score can range from 0 to 88. Sum scores of ≥24 indicate clinically meaningful post‐traumatic symptomatology and scores of ≥33 indicate probable post‐traumatic stress disorder (PTSD).^53^ Cronbach's alpha for the IES‐R total score was 0.92.

The 30‐item Life Stressor Checklist‐Revised (LSC‐R) is an index of lifetime trauma exposure (e.g., being mugged, sexual assault).^54^ The total LSC–R score is obtained by summing the total number of events endorsed (range of 0 to 30). If the patient endorsed an event, the patient was asked to indicate how much that stressor affected their life in the past year, from 1 (not at all) to 5 (extremely). These responses were summed to yield a total “affected” sum score. In addition, a PTSD sum score was created based on the number of positively endorsed items (out of 21) that reflect the DSM‐IV PTSD Criteria A for having experienced a traumatic event.

The 10‐item Connor‐Davidson Resilience Scale (CDRS) evaluates a patient's personal ability to handle adversity (e.g., “I am able to adapt when changes occur”).^55^, ^56^ Items are scored on a 5‐point Likert scale (“not true at all” to “true nearly all of the time”). Total scores range from 0 to 40, with higher scores indicative of higher self‐perceived resilience. The normative adult mean score in the United States is 31.8 (standard deviation [SD], 5.4),^56^, ^57^ with an estimated minimal clinically important difference of 2.7.^58^ Its Cronbach's alpha was 0.90.

### 2.4 Data analysis

Descriptive statistics and frequency distributions were generated for sample characteristics at enrollment using IBM SPSS Statistics version 29 (IBM Corporation, Armonk, NY). As was done previously,^59^ unconditional LPAs were used to identify distinct shortness of breath severity profiles, distress profiles, and joint shortness of breath severity AND distress profiles that characterized unobserved subgroups of patients (i.e., latent classes) over the six assessments. Three separate LPAs were performed using the available number of patients for each dimension AND joint dimensions of the symptom experience using MPlus™ Version 8.4.^60^

For each LPA, estimation was carried out with full information maximum likelihood with standard error and a Chi square test that are robust to non‐normality and nonindependence of observations (“estimator = MLR”). Model fit was evaluated to identify the solution that best characterized the observed latent class structure with the Bayesian information criterion (BIC), Vuong‐Lo–Mendell–Rubin likelihood ratio test (VLMR), entropy, and latent class percentages that were large enough to be reliable.^61^ Missing data were accommodated for with the use of the Expectation–Maximization (EM) algorithm.^62^

Differences among the latent classes in demographic, clinical, symptom, stress, and resilience characteristics were evaluated using parametric and nonparametric tests. A *p*‐value of <0.05 was considered statistically significant.

## 3 RESULTS

### 3.1 Latent profiles for the severity

For the severity of shortness of breath, a two‐class solution was selected because the BIC for that solution was lower than the BIC for the one‐class solution (Table 1). In addition, the VLMR was significant for the two‐class solution, indicating that two classes fit the data better than one class. The profiles were named based on an evaluation of the severity ratings over the six assessments, namely: Slight to Moderate (S‐M‐Severity; 91.6%) and Moderate to Severe (M‐S‐Severity; 8.4%).

**TABLE 1:** Latent profile solutions and fit indices for one through three classes for the Shortness of Breath Severity and Distress Scales on the Memorial Symptom Assessment Scale.

Model	LL	AIC	BIC	Entropy	VLMR
Severity scale					
1 Class	−1441.27	2916.53	2983.56	n/a	n/a
2 Class^a^	−1345.59	2739.18	2833.81	0.89	191.35*
3 Class	−1110.52	2283.04	2405.27	0.77	ns
Distress scale					
1 Class	−1932.92	3899.84	3966.83	n/a	n/a
2 Class^a^	−1810.91	3669.81	3764.37	0.87	244.03^†^
3 Class	−1783.24	3628.47	3750.62	0.76	ns
Severity and distress scales					
1 Class	−3378.85	6825.69	6959.92	n/a	n/a
2 Class^a^	−3092.45	6278.89	6464.45	0.87	572.80*
3 Class	−2834.05	5788.10	6024.98	0.85	ns

*Note*: Baseline entropy and VLMR are not applicable for the one‐class solution.

Abbreviations: AIC, Akaike's information criterion; BIC, Bayesian information criterion; LL, log‐likelihood; n/a, not applicable; ns, not significant; VLMR, Vuong‐Lo–Mendell–Rubin likelihood ratio test for the K versus K‐1 model.

^a^ For both the severity and distress scales, the two‐class solution was selected because the BIC was lower than the BIC for the one‐class solution. In addition, the VLMR was significant for the two‐class solution, indicating that two classes fit the data better than one class. Although the BIC for the three‐class solution was smaller than the BIC for the two‐class solution, the VLMR was not significant for the three‐class solution, indicating that too many classes had been extracted.

*p < 0.05, ^†^ *p* < 0.00005.

**FIGURE 1:**
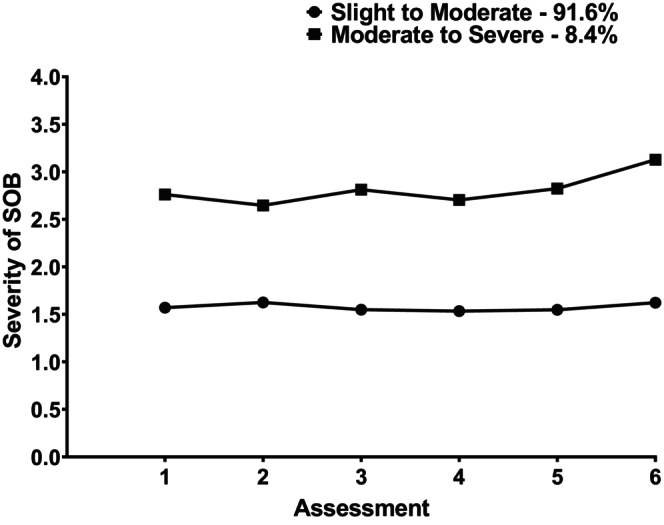
Changes in severity ratings for shortness of breath (SOB) over two cycles of chemotherapy for subgroups of patients with Slight to Moderate and Moderate to Severe ratings.

Figure 1 displays the trajectories of the severity ratings for the two profiles. For the S‐M‐Severity profile, severity ratings remained relatively low over the six assessments. For the M‐S‐Severity profile, while slightly higher ratings were reported at Assessment 6, the severity ratings remained consistently in the moderate to severe range.

### 3.2 Latent profiles for distress

For the distress from shortness of breath, a two‐class solution was selected because the BIC for that solution was lower than the BIC for the one‐class solution (Table 1). In addition, the VLMR was significant for the two‐class solution, indicating that two classes fit the data better than one class. Distress profiles were named based on an evaluation of the distress ratings over the six assessments, namely: A Little Bit to Somewhat (LB‐S‐Distress; 83.9%) and Somewhat to Quite a Bit (S‐QB‐Distress; 16.1%).

**FIGURE 2:**
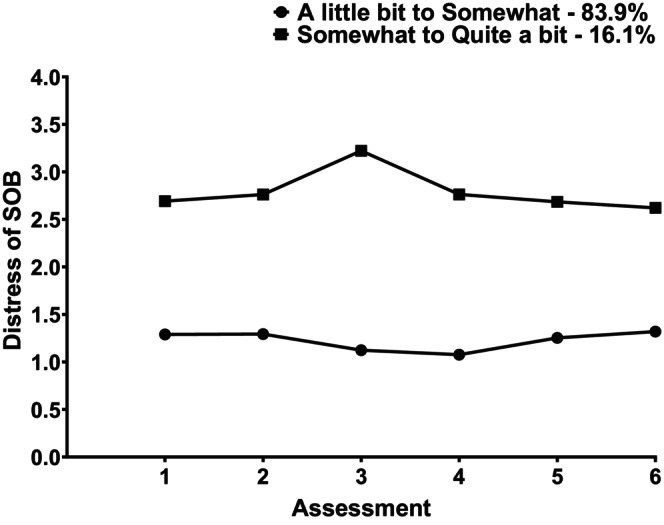
Changes in the distress ratings for shortness of breath (SOB) over two cycles of chemotherapy for patients with A little Bit to Somewhat and Somewhat to Quite a bit ratings.

Figure 2 displays the trajectories of the distress ratings for the two profiles. For the LB‐S‐Distress profile, while slightly lower distress ratings were reported at Assessments 3 and 4, the distress ratings remained in the little bit to somewhat range over the six assessments. For the S‐QB‐Distress profile, while slightly higher distress ratings were reported at Assessment 3, the distress ratings remained in the Somewhat to Quite a Bit range across the six assessments.

### 3.3 Joint LPA for severity AND distress

For the joint LPA of severity AND distress ratings of shortness of breath, a two‐class solution was selected because the BIC for that solution was lower than the BIC for the one‐class solution (Table 1). In addition, the VLMR was significant for the two‐class solution, indicating that two classes fit the data better than one class. The joint severity AND distress profiles were named based on and evaluation of the two symptom dimensions over the six assessments, namely: Lower Severity and Distress (Both Low, 79.9%) and Higher Severity and Distress (Both High, 20.1%).

**FIGURE 3:**
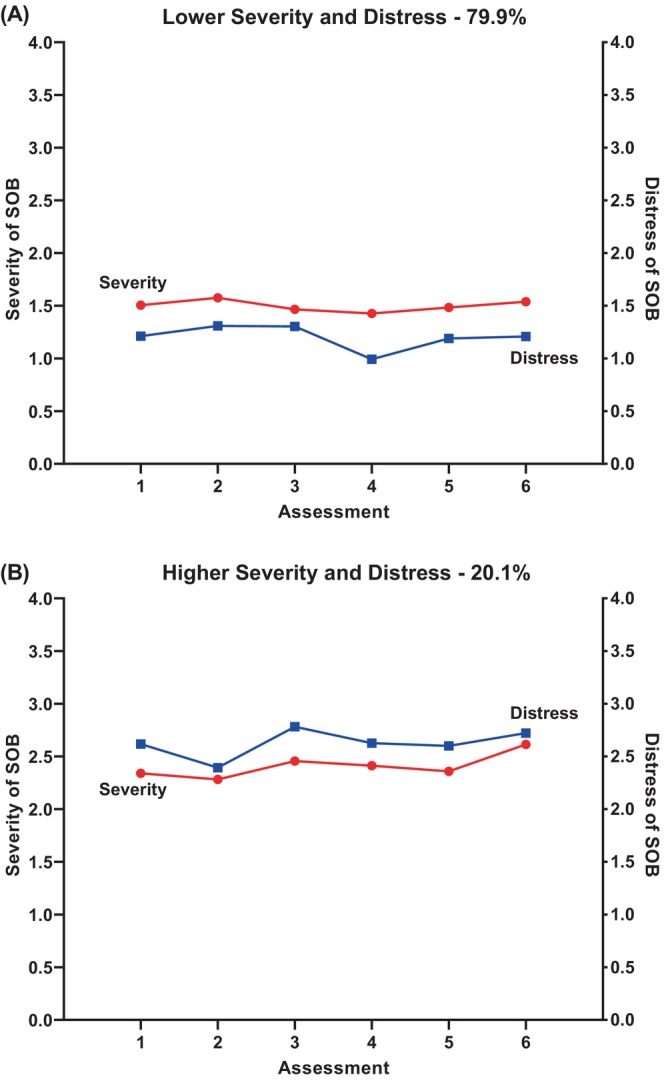
Changes in severity (left *y*‐axis) and distress (right *y*‐axis) ratings for shortness of breath (SOB) over two cycles of chemotherapy for subgroups of patients with Lower Severity and Distress (panel A) and Higher Severity and Distress (panel B).

Figure 3 displays the trajectories of the severity AND distress ratings for the two joint profiles. For the Both Low profile, the severity ratings remained in the slight to moderate range across the six assessments. While slightly lower distress ratings were reported at Assessment 4 (prior to chemotherapy administration), they remained relatively consistent in the little bit to somewhat range over the six assessments. For the Both High profile, while the severity ratings increased slightly at Assessments 3 and 6, they remained in the moderate to severe range across the six assessments. While the distress ratings decreased at Assessment 2 and increased at Assessment 3, they remained relatively consistent in the Somewhat to Quite a Lot range from Assessments 3 through 6.

### 3.4 Demographic and clinical characteristics

#### 3.4.1 Severity profiles

Compared to the S‐M‐Severity class, the M‐S‐Severity class had lower KPS scores, a higher number of comorbidities, and higher SCQ scores (Table 2). In addition, they were more likely to have a past or current history of smoking and self‐reported diagnoses of kidney disease and rheumatoid arthritis. No differences in demographic characteristics were found between the two classes.

**TABLE 2:** Differences in demographic and clinical characteristics at enrollment between the distinct shortness of breath severity profiles.

Characteristic	Slight to moderate, 91.6% (*n* = 349)	Moderate to severe, 8.4% (*n* = 32)	Statistics
	Mean (SD)	Mean (SD)	
Age (years)	57.2 (12.4)	61.3 (13.1)	*t* = −1.80, *p* = 0.073
Education (years)	16.1 (3.0)	15.6 (2.8)	*t* = 0.94, *p* = 0.350
Body mass index (kilogram/meter squared)	26.7 (6.2)	28.8 (7.1)	*t* = −1.83, *p* = 0.068
Alcohol use disorders identification test score	2.6 (2.2)	4.1 (4.0)	*t* = −1.46, *p* = 0.165
Karnofsky Performance Status score	76.6 (12.3)	69.3 (12.9)	*t* = 3.07, *p* = 0.002
Number of comorbid conditions	2.8 (1.6)	3.5 (1.9)	*t* = −2.54, *p* = 0.011
Self‐administered comorbidity questionnaire score	6.4 (3.6)	8.7 (5.0)	*t* = −2.58, *p* = 0.014
Time since diagnosis (years)	2.7 (4.9)	2.7 (6.9)	U, *p* = 0.255
Time since diagnosis (years, median)	0.48	0.37	
Number of prior cancer treatments	1.8 (1.7)	1.8 (1.5)	*t* = −0.07, *p* = 0.943
Number of metastatic sites including lymph node involvement^a^	1.3 (1.3)	1.3 (1.4)	*t* = 0.70, *p* = 0.944
Number of metastatic sites excluding lymph node involvement	0.9 (1.1)	0.9 (1.2)	*t* = −0.20, *p* = 0.842
MAX2 score	0.18 (0.08)	0.18 (0.08)	*t* = −0.31, *p* = 0.757
	% (*n*)	% (*n*)	
Gender (% female)	85.1 (297)	78.1 (25)	FE, *p* = 0.307
Self‐reported ethnicity			
White	71.4 (245)	67.7 (21)	*Χ* ^2^ = 1.07, *p* = 0.785
Asian or Pacific Islander	10.8 (37)	9.7 (3)	
Black	7.6 (26)	6.5 (2)	
Hispanic, mixed, or other	10.2 (35)	16.1 (5)	
Married or partnered (% yes)	58.7 (202)	65.6 (21)	FE, *p* = 0.573
Lives alone (% yes)	25.5 (88)	31.3 (10)	FE, *p* = 0.528
Currently employed (% yes)	30.3 (105)	21.9 (7)	FE, *p* = 0.419
Annual household income			
Less than $30,000	24.4 (76)	43.3 (13)	U, *p* = 0.207
$30,000–$70,000	22.1 (69)	10.0 (3)	
$70,000–$100,000	16.7 (52)	13.3 (4)	
Greater than $100,000	36.9 (115)	33.3 (10)	
Child care responsibilities (% yes)	19.5 (66)	33.3 (10)	FE *p* = 0.097
Elder care responsibilities (% yes)	8.9 (28)	15.4 (4)	FE, p = 0.289
Past or current history of smoking (% yes)	38.5 (132)	70.0 (21)	FE, *p* = 0.002
Exercise on a regular basis (% yes)	66.1 (222)	54.8 (17)	FE, *p* = 0.239
Specific comorbid conditions (% yes)			
Heart disease	6.9 (24)	12.5 (4)	FE, *p* = 0.277
High blood pressure	30.9 (108)	34.4 (11)	FE, *p* = 0.693
Lung disease	19.5 (68)	31.3 (10)	FE, *p* = 0.166
Diabetes	10.3 (36)	12.5 (4)	FE, *p* = 0.761
Ulcer or stomach disease	5.4 (19)	12.5 (4)	FE, *p* = 0.116
Kidney disease	1.7 (6)	9.4 (3)	FE, *p* = 0.032
Liver disease	7.7 (27)	6.3 (2)	FE, *p* = 1.000
Anemia or blood disease	16.9 (59)	25.0 (8)	FE, *p* = 0.234
Depression	28.1 (98)	34.4 (11)	FE, *p* = 0.540
Osteoarthritis	16.6 (58)	21.9 (7)	FE, *p* = 0.462
Back pain	33.2 (116)	43.8 (14)	FE, *p* = 0.246
Rheumatoid arthritis	3.2 (11)	12.5 (4)	FE, *p* = 0.029
Cancer diagnosis			
Breast cancer	45.8 (160)	31.3 (10)	*Χ* ^2^ = 3.41, *p* = 0.332
Gastrointestinal cancer	18.6 (65)	25.0 (8)	
Gynecological cancer	16.9 (59)	15.6 (5)	
Lung cancer	18.6 (65)	28.1 (9)	
Prior cancer treatment			
No prior treatment	23.2 (79)	25.8 (8)	*Χ* ^2^ = 4.00, *p* = 0.262
Only surgery, CTX, or RT	39.3 (134)	22.6 (7)	
Surgery and CTX, or surgery and RT, or CTX and RT	18.5 (63)	29.0 (9)	
Surgery and CTX and RT	19.1 (65)	22.6 (7)	
Receipt of targeted therapy (% yes)	36.2 (124)	37.5 (12)	FE, *p* = 0.850
Cycle length			
14‐day cycle	35.0 (121)	22.6 (7)	U, *p* = 0.086
21‐day cycle	56.6 (196)	61.3 (19)	
28‐day cycle	8.4 (29)	16.1 (5)	
Metastatic sites			
No metastasis	32.4 (112)	37.5 (12)	*Χ* ^2^ = 0.62, *p* = 0.891
Only lymph node metastasis	19.1 (66)	18.8 (6)	
Only metastatic disease in other sites	20.8 (72)	21.9 (7)	
Metastatic disease in lymph nodes and other sites	27.7 (96)	21.9 (7)	
Emetogenicity of the CTX regimen			
Minimal/low	23.3 (81)	22.6 (7)	U, *p* = 0.711
Moderate	55.0 (191)	61.3 (19)	
High	21.6 (75)	16.1 (5)	
Antiemetic regimen			
None	6.8 (23)	9.7 (3)	*Χ* ^2^ = 0.36, *p* = 0.949
Steroid alone or serotonin receptor antagonist alone	22.8 (77)	22.6 (7)	
Serotonin receptor antagonist and steroid	43.9 (148)	41.9 (13)	
NK‐1 receptor antagonist and two other antiemetics	26.4 (89)	25.8 (8)	

Abbreviations: CTX, chemotherapy; FE, Fisher's exact test; NK‐1, neurokinin‐1; NS, not significant; RT, radiation therapy; SD, standard deviation; U, Mann–Whitney *U* test.

^a^ Total number of metastatic sites evaluated was 9.

#### 3.4.2 Distress profiles

Compared to the LB‐S‐Distress class, the S‐QB‐Distress class had lower KPS scores, a higher number of comorbidities, and higher SCQ scores (Table 3). In addition, they were more likely to have a past or current history of smoking, self‐reported a diagnosis of lung disease, and were more likely to have received surgery and chemotherapy, surgery and radiation therapy, or chemotherapy and radiation therapy prior to this course of chemotherapy. No differences in demographic characteristics were found between the two classes.

**TABLE 3:** Differences in demographic and clinical characteristics at enrollment between the distinct shortness of breath distress profiles.

Characteristic	Little Bit to Somewhat, 83.9% (*n* = 319)	Somewhat to Quite a Bit, 16.1% (*n* = 61)	Statistics
	Mean (SD)	Mean (SD)	
Age (years)	57.2 (12.5)	59.3 (12.2)	*t* = −1.22, *p* = 0.223
Education (years)	16.0 (2.9)	16.2 (3.4)	*t* = −0.58, *p* = 0.560
Body mass index (kilogram/meter squared)	27.0 (6.5)	26.1 (5.2)	*t* = 0.99, *p* = 0.322
Alcohol use disorders identification test score	2.7 (2.4)	2.6 (2.4)	*t* = 0.28, *p* = 0.779
Karnofsky Performance Status score	76.9 (12.3)	71.4 (12.8)	*t* = 3.06, *p* = 0.002
Number of comorbid conditions	2.8 (1.6)	3.3 (1.7)	*t* = −2.42, *p* = 0.016
Self‐administered comorbidity questionnaire score	6.3 (3.6)	8.0 (4.3)	*t* = −3.31, *p* = 0.001
Time since diagnosis (years)	2.7 (2.4)	2.6 (2.4)	U, *p* = 0.808
Time since diagnosis (years, median)	0.48	0.46	
Number of prior cancer treatments	1.8 (1.7)	1.9 (1.6)	*t* = −0.38, *p* = 0.706
Number of metastatic sites including lymph node involvement^a^	1.3 (1.3)	1.4 (1.4)	*t* = −0.74, *p* = 0.460
Number of metastatic sites excluding lymph node involvement	0.8 (1.1)	1.1 (1.2)	*t* = −1.39, *p* = 0.116
MAX2 score	0.18 (0.09)	0.17 (0.08)	*t* = 0.30, *p* = 0.762
	% (*n*)	% (*n*)	
Gender (% female)	85.3 (272)	80.3 (49)	FE, *p* = 0.337
Self‐reported ethnicity			
White	70.5 (220)	73.8 (45)	*Χ* ^2^ = 1.73, *p* = 0.630
Asian or Pacific Islander	11.5 (36)	6.6 (4)	
Black	7.7 (24)	6.6 (4)	
Hispanic, mixed, or other	10.3 (32)	13.1 (8)	
Married or partnered (% yes)	57.8 (182)	65.0 (39)	FE, *p* = 0.319
Lives alone (% yes)	25.6 (81)	28.3 (17)	FE, *p* = 0.635
Currently employed (% yes)	30.6 (97)	25.0 (15)	FE, *p* = 0.443
Annual household income			
Less than $30,000	23.9 (68)	36.8 (21)	U, *p* < 0.539
$30,000–$70,000	23.2 (66)	10.5 (6)	
$70,000–$100,000	16.8 (48)	14.0 (8)	
Greater than $100,000	36.1 (103)	38.6 (22)	
Child care responsibilities (% yes)	21.4 (66)	18.6 (11)	FE *p* = 0.729
Elder care responsibilities (% yes)	10.6 (30)	3.7 (2)	FE, *p* = 0.134
Past or current history of smoking (% yes)	36.7 (115)	62.7 (37)	FE, *p* < 0.001
Exercise on a regular basis (% yes)	67.1 (206)	55.0 (33)	FE, *p* = 0.077
Specific comorbid conditions (% yes)			
Heart disease	7.5 (24)	6.6 (4)	FE, *p* = 1.000
High blood pressure	31.3 (100)	31.1 (19)	FE, *p* = 1.000
Lung disease	17.9 (57)	36.1 (22)	FE, *p* = 0.003
Diabetes	11.6 (37)	8.2 (5)	FE, *p* = 0.512
Ulcer or stomach disease	5.0 (16)	11.5 (7)	FE, *p* = 0.073
Kidney disease	2.2 (7)	3.3 (2)	FE, *p* = 0.641
Liver disease	7.5 (24)	6.6 (4)	FE, *p* = 1.000
Anemia or blood disease	16.0 (51)	26.2 (16)	FE, *p* = 0.066
Depression	26.6 (85)	39.3 (24)	FE, *p* = 0.063
Osteoarthritis	16.9 (54)	18.0 (11)	FE, *p* = 0.853
Back pain	32.6 (104)	42.6 (26)	FE, *p* = 0.142
Rheumatoid arthritis	4.1 (13)	3.3 (2)	FE, *p* = 1.000
Cancer diagnosis			
Breast cancer	46.1 (147)	36.1 (22)	*Χ* ^2^ = 3.99, *p* = 0.262
Gastrointestinal cancer	18.5 (59)	21.3 (13)	
Gynecological cancer	17.2 (55)	14.8 (9)	
Lung cancer	18.2 (58)	27.9 (17)	
Prior cancer treatment			*Χ* ^2^ = 12.38, *p* = 0.006
No prior treatment	22.6 (70)	26.2 (16)	NS
Only surgery, CTX, or RT	41.3 (128)	21.3 (13)	1 > 2
Surgery and CTX, or surgery and RT, or CTX and RT	16.8 (52)	32.8 (20)	1 < 2
Surgery and CTX and RT	19.4 (60)	19.7 (12)	NS
Receipt of targeted therapy (% yes)	34.5 (108)	45.9 (28)	FE, *p* = 0.109
Cycle length			
14‐day cycle	34.7 (110)	27.1 (16)	U, *p* = 0.146
21‐day cycle	57.1 (181)	59.3 (35)	
28‐day cycle	8.2 (26)	13.6 (8)	
Metastatic sites			
No metastasis	32.6 (103)	32.8 (20)	*Χ* ^2^ = 3.15, *p* = 0.370
Only lymph node metastasis	20.6 (216)	13.1 (8)	
Only metastatic disease in other sites	19.6 (62)	27.9 (17)	
Metastatic disease in lymph nodes and other sites	27.2 (86)	26.2 (16)	
Emetogenicity of the CTX regimen			
Minimal/low	23.6 (75)	23.7 (14)	U, *p* = 0.257
Moderate	53.8 (171)	64.4 (38)	
High	22.6 (72)	11.9 (7)	
Antiemetic regimen			
None	6.2 (19)	10.2 (6)	*Χ* ^2^ = 1.96, *p* = 0.581
Steroid alone or serotonin receptor antagonist alone	24.0 (74)	18.6 (11)	
Serotonin receptor antagonist and steroid	43.8 (135)	42.4 (25)	
NK‐1 receptor antagonist and two other antiemetics	26.0 (80)	28.8 (17)	

Abbreviations: CTX, chemotherapy; FE, Fisher's exact test; NK‐1, neurokinin‐1; NS, not significant; RT, radiation therapy; SD, standard deviation; U, Mann–Whitney *U* test.

^a^ Total number of metastatic sites evaluated was 9.

#### 3.4.3 Joint profiles

Compared to the Both Low class, Both High class had lower KPS scores, a higher number of comorbidities, and higher SCQ scores (Table 4). They were more likely to have a past or current history of smoking, self‐reported diagnoses of lung disease, ulcer or stomach disease, depression, and back pain. In addition, Both High class was more likely to have received surgery and chemotherapy, surgery and radiation therapy, or chemotherapy and radiation therapy prior to this course of chemotherapy. No differences in demographic characteristics were found between the two classes.

**TABLE 4:** Differences in demographic and clinical characteristics at enrollment between the distinct shortness of breath severity and distress joint profiles.

Characteristic	Lower Severity and Distress, 79.9% (*n* = 306)	Higher Severity and Distress, 20.1% (*n* = 77)	Statistics
	Mean (SD)	Mean (SD)	
Age (years)	57.2 (12.6)	59.0 (12.0)	*t* = −1.16, *p* = 0.245
Education (years)	16.1 (2.9)	15.9 (3.3)	*t* = 0.45, *p* = 0.651
Body mass index (kilogram/meter squared)	27.0 (6.4)	26.4 (6.0)	*t* = 0.67, *p* = 0.505
Alcohol use disorders identification test score	2.7 (2.3)	2.9 (2.9)	*t* = −0.62, *p* = 0.534
Karnofsky Performance Status score	76.9 (12.3)	71.7 (12.9)	*t* = 3.13, *p* = 0.002
Number of comorbid conditions	2.7 (1.6)	3.3 (1.7)	*t* = −2.92, *p* = 0.004
Self‐administered comorbidity questionnaire score	6.2 (3.6)	8.0 (4.2)	*t* = −3.72, *p* < 0.001
Time since diagnosis (years)	2.6 (5.0)	2.8 (5.6)	U, *p* = 0.906
Time since diagnosis (years, median)	0.47	0.50	
Number of prior cancer treatments	1.8 (1.6)	2.1 (1.8)	*t* = −1.37, *p* = 0.173
Number of metastatic sites including lymph node involvement^a^	1.3 (1.3)	1.5 (1.4)	*t* = −1.26, *p* = 0.210
Number of metastatic sites excluding lymph node involvement	0.8 (1.1)	1.1 (1.2)	*t* = −1.65, *p* = 0.101
MAX2 score	0.17 (0.09)	0.18 (0.08)	*t* = −0.81, *p* = 0.421
	% (*n*)	% (*n*)	
Gender (% female)	85.3 (261)	81.8 (63)	FE, *p* = 0.480
Self‐reported ethnicity			
White	70.8 (213)	72.0 (54)	*Χ* ^2^ = 1.40, *p* = 0.706
Asian or Pacific Islander	11.3 (34)	8.0 (6)	
Black	8.0 (24)	6.7 (5)	
Hispanic, mixed, or other	10.0 (30)	13.3 (10)	
Married or partnered (% yes)	58.3 (176)	61.8 (47)	FE, *p* = 0.604
Lives alone (% yes)	24.8 (75)	30.3 (23)	FE, *p* = 0.379
Currently employed (% yes)	30.9 (94)	23.7 (18)	FE, *p* = 0.261
Annual household income			
Less than $30,000	22.6 (62)	41.4 (29)	U, *p* = 0.078
$30,000–$70,000	23.7 (65)	10.0 (7)	
$70,000–$100,000	16.4 (45)	15.7 (11)	
Greater than $100,000	37.2 (102)	32.9 (23)	
Child care responsibilities (% yes)	20.0 (59)	24.0 (18)	FE *p* = 0.431
Elder care responsibilities (% yes)	9.9 (27)	7.2 (5)	FE, *p* = 0.646
Past or current history of smoking (% yes)	36.7 (110)	58.7 (44)	FE, *p* < 0.001
Exercise on a regular basis (% yes)	66.7 (196)	57.3 (43)	FE, *p* = 0.138
Specific comorbid conditions (% yes)			
Heart disease	7.5 (23)	6.5 (5)	FE, *p* = 1.000
High blood pressure	32.0 (98)	28.6 (22)	FE, *p* = 0.586
Lung disease	17.6 (54)	32.5 (25)	FE, *p* = 0.007
Diabetes	11.4 (35)	9.1 (7)	FE, *p* = 0.685
Ulcer or stomach disease	4.6 (14)	11.7 (9)	FE, *p* = 0.029
Kidney disease	2.0 (6)	3.9 (3)	FE, *p* = 0.393
Liver disease	7.2 (22)	9.1 (7)	FE, *p* = 0.629
Anemia or blood disease	15.7 (48)	24.7 (19)	FE, *p* = 0.067
Depression	25.2 (77)	42.9 (33)	FE, *p* = 0.003
Osteoarthritis	16.7 (51)	18.2 (14)	FE, *p* = 0.736
Back pain	31.7 (97)	44.2 (34)	FE, *p* = 0.044
Rheumatoid arthritis	3.9 (12)	3.9 (3)	FE, *p* = 1.000
Cancer diagnosis			
Breast cancer	45.8 (140)	40.3 (31)	*Χ* ^2^ = 2.67, *p* = 0.446
Gastrointestinal cancer	19.6 (60)	16.9 (13)	
Gynecological cancer	16.7 (51)	16.9 (13)	
Lung cancer	18.0 (55)	26.0 (20)	
Prior cancer treatment			*Χ* ^2^ = 8.19, *p* = 0.042
No prior treatment	23.2 (69)	23.7 (18)	NS
Only surgery, CTX, or RT	40.9 (122)	26.3 (20)	1 > 2
Surgery and CTX, or surgery and RT, or CTX and RT	16.8 (50)	28.9 (22)	1 < 2
Surgery and CTX and RT	19.1 (57)	21.1 (16)	NS
Receipt of targeted therapy (% yes)	34.7 (104)	42.9 (33)	FE, *p* = 0.187
Cycle length			
14‐day cycle	36.2 (110)	24.0 (18)	U, *p* = 0.082
21‐day cycle	54.9 (167)	66.7 (50)	
28‐day cycle	8.9 (27)	9.3 (7)	
Metastatic sites			
No metastasis	33.3 (101)	29.9 (23)	*Χ* ^2^ = 1.61, *p* = 0.657
Only lymph node metastasis	19.8 (60)	16.9 (13)	
Only metastatic disease in other sites	19.8 (60)	26.0 (20)	
Metastatic disease in lymph nodes and other sites	27.1 (82)	27.3 (21)	
Emetogenicity of the CTX regimen			
Minimal/low	23.9 (73)	22.7 (17)	U, *p* = 0.344
Moderate	53.1 (162)	64.0 (48)	
High	23.0 (70)	13.3 (10)	
Antiemetic regimen			
None	7.1 (21)	6.7 (5)	*Χ* ^2^ = 0.08, *p* = 0.994
Steroid alone or serotonin receptor antagonist alone	22.7 (67)	24.0 (18)	
Serotonin receptor antagonist and steroid	43.7 (129)	42.7 (32)	
NK‐1 receptor antagonist and two other antiemetics	26.4 (78)	26.7 (20)	

Abbreviations: CTX, chemotherapy; FE, Fisher's exact test; NK‐1, neurokinin‐1; NS, not significant; RT, radiation therapy; SD, standard deviation; U, Mann–Whitney *U* test.

^a^ Total number of metastatic sites evaluated was 9.

### 3.5 Symptom severity

Compared to the S‐M‐Severity class, the M‐S‐Severity class reported higher levels of depressive symptoms, trait and state anxiety, morning and evening fatigue, sleep disturbance, worst pain, and pain interference. In addition, they reported significant decrements in morning and evening energy and cognitive function (Table 5).

**TABLE 5:** Differences in co‐occurring symptom severity scores at enrollment between the distinct shortness of breath severity and distress profiles.

Symptoms^a^	Severity classes			Distress classes			Joint severity and distress classes		
	Slight to moderate, 91.6% (*n* = 349)	Moderate to severe, 8.4% (*n* = 32)	*p*‐value	Little Bit to Somewhat, 83.9% (*n* = 319)	Somewhat to Quite a Bit, 16.1% (*n* = 61)	*p*‐value	Lower Severity and Distress (1), 79.9% (*n* = 306)	Higher Severity and Distress (2), 20.1% (*n* = 77)	*p*‐value
	Mean (SD)	Mean (SD)		Mean (SD)	Mean (SD)		Mean (SD)	Mean (SD)	
Depressive symptoms (≥16)	15.2 (10.4)	20.5 (12.5)	*p* = 0.007	14.8 (10.1)	20.9 (12.3)	*p* < 0.001	14.7 (10.2)	20.0 (11.5)	*p* < 0.001
Trait anxiety (≥31.8)	37.1 (10.8)	43.7 (13.2)	*p* = 0.010	36.9 (10.7)	42.2 (13.1)	*p* = 0.006	36.9 (10.7)	40.9 (12.5)	*p* = 0.007
State anxiety (≥32.2)	35.5 (13.0)	44.5 (16.0)	*p* < 0.001	35.3 (12.7)	41.9 (16.8)	*p* = 0.006	35.2 (12.8)	41.0 (15.7)	*p* = 0.004
Morning fatigue (≥3.2)	3.8 (2.2)	5.5 (2.6)	*p* < 0.001	3.8 (2.2)	4.7 (2.3)	*p* = 0.004	3.8 (2.2)	4.6 (2.3)	*p* = 0.009
Evening fatigue (≥5.6)	5.7 (1.8)	6.8 (1.9)	*p* < 0.001	5.7 (1.8)	6.2 (2.1)	*p* = 0.044	5.7 (1.8)	6.3 (1.9)	*p* = 0.011
Morning energy (≤6.2)	4.1 (2.0)	2.9 (2.4)	*p* = 0.009	4.1 (2.0)	3.6 (2.0)	*p* = 0.006	4.1 (2.1)	3.5 (2.0)	*p* = 0.022
Evening energy (≤3.5)	3.5 (2.0)	2.1 (2.0)	*p* < 0.001	3.6 (2.0)	2.2 (1.7)	*p* < 0.001	3.6 (2.0)	2.5 (1.7)	*p* < 0.001
Sleep disturbance (≥43.0)	58.0 (19.1)	66.9 (21.0)	*p* = 0.014	57.0 (19.1)	68.1 (19.4)	*p* < 0.001	57.1 (19.1)	65.8 (19.6)	*p* < 0.001
Attentional function (<5.0 = Low, 5 to 7.5 = Moderate, >7.5 = High)	6.0 (1.7)	5.0 (1.9)	*p* = 0.002	6.0 (1.7)	5.5 (1.9)	*p* = 0.075	6.0 (1.7)	5.4 (1.7)	*p* = 0.010
	% (*n*)	% (*n*)		% (*n*)	% (*n*)		% (*n*)	% (*n*)	
Type of pain			*p* = 0.082			*p* = 0.088			*p* = 0.028
No pain	21.2 (73)	6.7 (2)		21.6 (68)	10.3 (6)		22.5 (68)	9.5 (7)	1 > 2
Only noncancer pain	29.1 (100)	20.0 (6)		12.4 (39)	10.3 (6)		28.8 (87)	25.7 (19)	NS
Only cancer pain	11.3 (39)	16.7 (5)		28.6 (90)	25.9 (15)		11.9 (36)	12.2 (9)	NS
Both cancer and noncancer pain	38.4 (132)	56.7 (17)		37.5 (118)	53.4 (31)		36.8 (111)	52.7 (39)	1 < 2
For patients with pain	Mean (SD)	Mean (SD)		Mean (SD)	Mean (SD)		Mean (SD)	Mean (SD)	
Worst pain score	6.4 (2.5)	7.6 (2.7)	*p* = 0.037	6.4 (2.5)	6.9 (2.8)	*p* = 0.234	6.4 (2.4)	7.2 (2.8)	*p* = 0.033
Mean pain interference score	3.7 (2.6)	5.2 (2.9)	*p* = 0.007	3.7 (2.6)	4.6 (2.7)	*p* = 0.017	3.6 (2.6)	4.7 (2.6)	*p* = 0.004

Abbreviation: SD, standard deviation.

^a^ Clinically meaningful cutoff scores.

Compared to the LB‐S‐Distress class, the S‐QB‐Distress class reported higher levels of depressive symptoms, trait and state anxiety, morning and evening fatigue, sleep disturbance, and pain interference. In addition, they reported significant decrements in morning and evening energy (Table 5).

Compared to the Both Low class, Both High class reported higher levels of depressive symptoms, trait and state anxiety, morning and evening fatigue, sleep disturbance, worst pain, and pain interference. In addition, they were more likely to have both cancer and noncancer pain and reported significant decrements in morning and evening energy and attentional function (Table 5).

### 3.6 Stress and resilience

Compared to the S‐M‐Severity class, the M‐S‐Severity class reported higher IES‐R total, IES‐R intrusion, and IES‐R hyperarousal scores. In terms of the PSS total, IES‐R avoidance, LSC‐R total, LSC‐R affected sum, LSC‐R PTSD sum, and CDRS total scores, no differences were found between the two classes (Table 6).

**TABLE 6:** Differences in stress and resilience measures at enrollment between the distinct shortness of breath severity and distress profiles.

Symptoms^a^	Severity Classes			Distress Classes			Joint Severity and Distress Classes		
	Slight to Moderate, 91.6% (*n* = 349)	Moderate to Severe, 8.4% (*n* = 32)	*p*‐value	Little Bit to Somewhat, 83.9% (*n* = 319)	Somewhat to Quite a Bit, 16.1% (*n* = 61)	*p*‐value	Lower Severity and Distress, 79.9% (*n* = 306)	Higher Severity and Distress, 20.1% (*n* = 77)	*p*‐value
	Mean (SD)	Mean (SD)		Mean (SD)	Mean (SD)		Mean (SD)	Mean (SD)	
PSS total score (range 0 to 56)	20.0 (8.2)	23.1 (8.8)	*p* = 0.053	19.7 (8.0)	23.6 (9.3)	*p* < 0.001	19.7 (8.1)	23.0 (8.7)	*p* = 0.002
IES‐R total score (≥24.0 – clinically meaningful PTSD symptomatology) (≥33.0 – probable PTSD)	21.2 (14.7)	30.7 (19.1)	*p* = 0.012	20.5 (14.3)	29.8 (19.0)	*p* < 0.001	20.5 (14.3)	28.6 (18.2)	*p* < 0.001
IES‐R intrusion	1.0 (0.8)	1.5 (1.0)	*p* = 0.002	1.0 (0.8)	1.5 (1.0)	*p* < 0.001	1.0 (0.8)	1.4 (1.0)	*p* = 0.001
IES‐R avoidance	1.0 (0.7)	1.2 (0.8)	*p* = 0.136	1.0 (0.7)	1.2 (0.8)	*p* = 0.012	1.0 (0.7)	1.2 (0.8)	*p* = 0.008
IES‐R hyperarousal	0.8 (0.7)	1.4 (1.0)	*p* = 0.005	0.8 (0.7)	1.2 (1.0)	*p* < 0.001	0.8 (0.7)	1.2 (1.0)	*p* = 0.002
LSC‐R total score (range 0–30)	6.9 (4.5)	7.7 (5.1)	*p* = 0.371	6.9 (4.4)	7.6 (5.2)	*p* = 0.165	6.7 (4.3)	8.0 (5.3)	*p* = 0.078
LSC‐R affected sum score (range 0–150)	13.9 (12.7)	19.0 (16.5)	*p* = 0.056	13.8 (12.4)	17.7 (16.3)	*p* = 0.058	13.2 (11.0)	19.2 (18.7)	*p* = 0.017
LSC‐R PTSD sum score (range 0–21)	3.7 (3.5)	4.6 (3.7)	*p* = 0.193	3.6 (3.5)	4.4 (3.9)	*p* = 0.075	3.5 (3.3)	4.8 (4.1)	*p* = 0.015
CDRS total score (range 0–40) (31.8 (±5.4) – normative mean score for the United States population)	29.7 (6.0)	28.1 (7.4)	*p* = 0.186	29.8 (6.0)	28.3 (6.6)	*p* = 0.055	29.8 (6.1)	28.3 (6.4)	*p* = 0.065

Abbreviations: CDRS, Connor Davidson Resilience Scale; IES‐R, Impact of Event Scale – Revised; LSC‐R, Life Stressor Checklist‐Revised; PSS, Perceived Stress Scale; PTSD, post‐traumatic stress disorder; SD, standard deviation.

^a^ Clinically meaningful cutoff scores or range of scores.

Compared to the LB‐S‐Distress class, the S‐QB‐Distress class reported higher PSS total, IES‐R total, IES‐R intrusion, IES‐R avoidance, and IES‐R hyperarousal scores. In terms of the LSC‐R total, LSC‐R affected sum, LSC‐R PTSD sum, and CDRS total scores, no differences were found between the two classes (Table 6).

Compared to the Both Low class, Both High class reported higher PSS total, IES‐R total, IES‐R intrusion, IES‐R avoidance, and IES‐R hyperarousal scores. In addition, they reported higher LSC‐R affected sum and LSC‐R PTSD sum scores. In terms of the LSC‐R total and CDRS total scores, no differences were found between the two classes (Table 6).

## 4 DISCUSSION

This study is the first to use LPA to identify subgroups of oncology patients with distinct shortness of breath severity and distress profiles, as well as a joint profile that used ratings of both severity and distress. In addition, this study is the first to evaluate for risk factors associated with higher levels of distress from shortness of breath. Of the 30% of patients in the total sample who reported shortness of breath,^33^ 8.4% reported severity ratings that ranged from moderate to severe;16.1% reported distress ratings that ranged from Somewhat to Quite a Bit; and 20.1% reported higher ratings of both severity and distress over two cycles of chemotherapy. The percentage of patients in the worse distress class is approximately double that of patients in the worse severity class. In addition, the proportion of patients in the Both High class was higher than the percentages of patients in the worse classes for severity or distress.

These findings have important clinical implications. An evaluation of the occurrence and/or severity of shortness of breath using a unidimensional scale is common in clinical practice.^63^ However, this approach will not identify patients with higher distress ratings and may underestimate the impact of shortness of breath on their lives. In addition, given that 20% of the patients with shortness of breath, regardless of their cancer diagnosis, reported relatively high severity and distress scores, supports the need for a multidimensional assessment of this symptom.

Given that the primary goal of this study was to identify common and distinct risk factors associated with more severe and distressing shortness of breath, these findings are summarized in Table 7. The remainder of the discussion places our findings in the context of the extant literature.

**TABLE 7:** Characteristics associated with membership in the worst classes for severity, distress, and joint severity and distress latent profile analyses.

Characteristic	Severity	Distress	Joint
	Moderate to Severe^a^	Somewhat to Quite a Bit^b^	Higher Severity and Distress^c^
Clinical characteristics			
More likely to have past or current history of smoking	■	■	■
Lower functional status	■	■	■
Higher number of comorbidities	■	■	■
Higher comorbidity burden	■	■	■
More likely to self‐report lung disease		■	■
More likely to self‐report kidney disease	■		
More likely to self‐report ulcer or stomach disease			■
More likely to self‐report depression			■
More likely to self‐report back pain			■
More likely to self‐report rheumatoid arthritis	■		
Less likely to have received only surgery, CTX, or RT		■	■
More likely to have received two of the following treatments: surgery, radiation, and CTX		■	■
Symptom characteristics			
Higher depressive symptoms	■	■	■
Higher trait anxiety	■	■	■
Higher state anxiety	■	■	■
Higher morning fatigue	■	■	■
Higher evening fatigue	■	■	■
Lower morning energy	■	■	■
Lower evening energy	■	■	■
Higher sleep disturbance	■	■	■
Lower attentional function	■		■
Less likely not to have pain			■
More likely to have both cancer and noncancer pain			■
Higher worst pain score	■		■
Higher mean pain interference score	■	■	■
Stress and Resilience Measures			
Higher Perceived Stress Scale score		■	■
Higher Impact of Event Scale‐Revised total score	■	■	■
Higher Impact of Event Scale‐Revised intrusion score	■	■	■
Higher Impact of Event Scale‐Revised avoidance score		■	■
Higher Impact of Event Scale‐Revised hyperarousal score	■	■	■
Higher Life Stressor Checklist‐Revised affected sum score			■
Higher Life Stressor Checklist‐Revised PTSD sum score			■

*Note*: Severity ratings – 1 = slight, 2 = moderate, 3 = severe, 4 = very severe.

Distress ratings – 0 = not at all, 1 = a little bit, 2 = somewhat, 3 = quite a bit, 4 = very much.

■ – Indicates that the class had this characteristic compared to the worst class in the severity, distress, and joint severity and distress latent profile analyses.

Abbreviations: CTX, chemotherapy; RT, radiation therapy; PTSD, post‐traumatic stress disorder.

^a^ Comparisons done with the slight to moderate class.

^b^ Comparisons done with the little bit to somewhat class.

^c^ Comparisons done with the lower severity and distress class.

### 4.1 Demographic characteristics

While no demographic characteristics were identified as risk factors for the worse shortness of breath profiles, in previous studies, older age, being male, lower education levels, lower income, and living alone were associated with higher occurrence rates^33^, ^64^ and severity ratings.^9^, ^10^, ^11^, ^16^, ^17^ Reasons for these inconsistent findings may be related to the relatively small number of patients in the worse classes and/or the use of a variety of measures to assess shortness of breath.

### 4.2 Clinical characteristics

#### 4.2.1 Common risk factors

Common risk factors associated with membership in the worse severity and distress classes across the three LPAs included: a past and current history of smoking, lower functional status, and higher comorbidity burden. Our findings are consistent with previous studies of oncology patients that found that a current history of smoking was associated with higher occurrence^33^ and severity^12^, ^65^ of shortness of breath. However, the current study provides new evidence of this characteristic being associated with higher levels of distress. In addition, it is congruent with previous findings that more frequent emotional problems^66^ and higher levels of lung cancer‐related symptom distress^65^ were reported by patients who used tobacco.

Consistent with studies of patients with advanced or non‐small cell lung cancer,^11^, ^13^, ^14^, ^15^, ^16^ a lower functional status was associated with more severe shortness of breath. While no associations were reported between distress and lower functional status, one plausible hypothesis for this finding is that lower functional status may interfere with daily activities and increase the unpleasantness from shortness of breath.^67^, ^68^ This hypothesis is supported by findings from studies of oncology outpatients with pain.^67^, ^68^ For example, in one study,^67^ higher levels of pain‐related distress predicted lower functional status and higher levels of interference with daily activities.

Across the three LPAs, membership in the worse classes was associated with a higher comorbidity burden. While previous reports found that a higher number of comorbid conditions^9^ and higher comorbidity burden^10^ were associated with more severe shortness of breath, no studies described this relationship with distress. One plausible explanation is that multimorbidity may increase severity and distress ratings by increasing overall symptom burden^69^ and emotional distress^70^ and decreasing functional status^71^ and quality of life.^72^ This hypothesis is consistent with our current findings across the three LPAs, that patients with the worse profiles reported lower KPS scores, higher levels of depressive symptoms and anxiety, and higher levels of other common symptoms.

#### 4.2.2 Distinct risk factors

Consistent with our a priori hypothesis, distinct clinical risk factors were identified across the three LPAs. For example, the occurrence of self‐reported kidney disease and rheumatoid arthritis was associated with membership only in the worse severity class. In contrast, the occurrence of self‐reported lung disease and the receipt of previous combination treatments were associated with membership in the worse distress and joint classes. In addition, patients in the worse severity AND distress class were more likely to self‐report ulcer or stomach disease, depression, and back pain.

While the prevalence of kidney disease in the M‐S‐Severity class was relatively low (9.4%), previous research found that 11%–55% of patients with chronic kidney disease report shortness of breath.^73^ In addition, in patients with chronic obstructive pulmonary disease (COPD),^74^ the occurrence of chronic kidney disease was associated with more severe shortness of breath. These associations may be related to anemia or concurrent cardiac problems (e.g., heart failure).^8^, ^75^

It is interesting to note that in a longitudinal study of patients with rheumatoid arthritis,^76^ 13.5% developed shortness of breath on exertion over a 3‐year period. In this study,^76^ the most significant predictor of moderate to severe shortness of breath was a lower functional status after controlling for age, sex, smoking history, and cardiopulmonary comorbidity.

While previous studies found associations between the occurrence of lung disease and higher occurrence^33^, ^64^ and severity^11^, ^12^, ^14^, ^77^ of shortness of breath, in the current study, associations were found only in patients in the worse distress and joint classes. One reason for the lack of significant findings for the severity LPA is the relatively small number of patients in the M‐S‐Severity class. In terms of distress, for patients with cancer, the occurrence of lung comorbidity may have a negative impact on their cognitive appraisal of the long‐term implications of having shortness of breath.^78^, ^79^ This hypothesis is supported by findings from a study that compared lung cancer patients with and without COPD and found that those with COPD reported a higher respiratory symptom burden and higher levels of activity avoidance.^80^

While no studies evaluated for associations between distress and types and number of previous cancer treatment(s), one plausible explanation for this finding is that cancer and its treatments are associated with high levels of emotional distress.^81^, ^82^ In terms of severity, our findings are consistent with a previous study that noted that the type of prior cancer treatment(s) in patients with lung cancer was not associated with the severity of shortness of breath.^12^

It is interesting to note that the Both High class reported higher rates of ulcer or stomach disease, depression, and back pain. While ulcer or stomach disease was reported by 11.7% of the patients in this class, no studies were identified that reported an association with shortness of breath. In terms of back pain, in a study of Medicare recipients,^83^ 50% of the adults who reported shortness of breath noted the co‐occurrence of musculoskeletal pain, including chronic back pain. While multiple mechanisms may explain this relationship, pain, physical deconditioning, and poorer functional status associated with back pain may contribute to higher ratings of both the severity and distress from shortness of breath.^84^

In terms of depression, 42.9% of patients in the Both High class self‐reported a diagnosis of depression. This finding is consistent with an Australian population‐based study that found that the occurrence of depression was the most significant predictor of more severe shortness of breath after controlling for age, sex, and functionality.^85^ In a study of patients with lung cancer,^21^ compared to those without depression, those with depression reported higher total, physical, and emotional scores on the Dyspnea‐12 questionnaire. In another study,^86^ the occurrence of depression was positively correlated with the Cancer Dyspnea Scale's sensory perceptual (i.e., the sense of breathing effort and the sense of breathing discomfort) and affective distress (i.e., the sense of anxiety) subscale scores. These findings suggest that the presence of depression may influence both the severity and distress of shortness of breath.

### 4.3 Common cancer‐related symptoms

#### 4.3.1 Common risk factors

Across all three LPAs (i.e., severity, distress and joint severity AND distress), patients with the worse profiles reported higher levels of depressive symptoms, trait and state anxiety, morning and evening fatigue, sleep disturbance, and pain interference. In addition, they reported significant decrements in morning and evening energy.

Patients in all three of the worse classes reported clinically meaningful levels of depressive symptoms. This finding is not surprising given that in previous studies of patients with advanced cancer,^11^, ^19^ higher depression scores were significant predictors of more severe shortness of breath. In addition, in a previous study of patients with lung cancer,^3^ a moderate positive correlation was found between distress ratings for shortness of breath and the severity of depressive symptoms. Across the three LPAs, regardless of class membership, 36.3%–52.6% of the patients reported CES‐D scores above the clinically meaningful cutpoint. These occurrence rates are higher than the 9.2% reported in a nationally representative study of the United States general population^87^ and the 27% reported in a meta‐analysis of depressive symptoms in oncology patients.^88^

Consistent with previous findings,^3^ patients in the three worse classes had higher levels of state and trait anxiety. Across these three classes, 60.7%–73.3% and 73.2%–75% of patients had clinically meaningful levels of state and trait anxiety, respectively. These occurrence rates are consistent with the 72% reported by critically ill patients with shortness of breath.^89^ Taken together, these findings suggest that shortness of breath increases anxiety and depression or visa versa.^90^

Consistent with previous findings,^19^, ^24^, ^25^, ^26^ across all three LPAs, patients with the worse profiles reported higher levels of morning and evening fatigue. Several hypotheses may explain these relationships. Patients with shortness of breath often limit their physical activity to avoid unpleasantness and distress from shortness of breath.^91^, ^92^ Constant sedentary lifestyles, systemic inflammation,^93^ and physical deconditioning^94^ may increase fatigue and decrease energy levels. This hypothesis is supported by studies of outpatients with advanced lung cancer^95^ and patients with COPD^96^ that demonstrated increased interference with physical activities was associated with increases in the severity of both shortness of breath and fatigue.

Consistent with previous studies in oncology patients,^4^, ^19^, ^97^ as well as in patients with asthma,^98^ COPD,^98^, ^99^ COVID‐19,^100^ and heart failure,^101^ patients with the three worse profiles reported higher levels of sleep disturbance. Across the three LPAs, regardless of class membership, 75.5%–89.8% of the patients reported GSDS scores above the clinically meaningful cutpoint. These occurrence rates are higher than the 60.7% reported in a meta‐analysis of sleep disturbance in patients with cancer.^102^ During sleep, changes occur in respiratory muscle function and ventilatory control.^103^ Muscle weakness and ventilatory imbalance associated with shortness of breath may contribute to decreases in sleep quality and duration.^98^, ^100^ In addition, the association between shortness of breath and sleep disturbance may be mediated by other co‐occurring symptoms.^59^ For example, in a study of patients with COVID‐19,^100^ anxiety mediated the relationship between sleep disturbance and the physical and affective aspects of shortness of breath.

Higher pain interference scores were reported by patients in the three worse classes. These findings are consistent with a previous report that noted that 30% of advanced lung cancer patients with shortness of breath and/or pain reported higher interference scores in daily activities.^95^

#### 4.3.2 Distinct risk factors

Consistent with studies of oncology patients,^4^, ^11^, ^13^, ^25^ membership in the M‐S‐Severity and the Both High classes was associated with more severe pain. In addition, the Both High class was more likely to have both cancer and noncancer pain. Of note, the pain intensity scores across all three LPAs were in the severe range. While no studies have evaluated for associations between pain intensity and distress ratings for shortness of breath, severe pain may increase its unpleasantness by worsening mood and decreasing enjoyment from daily activities.^95^ While clinical guidelines for shortness of breath^63^ and pain^104^ recommend the use of opioids to decrease each symptom, in two meta‐analyses,^105^, ^106^ findings on the efficacy of opioids to relieve shortness of breath were inconclusive. Of note, the occurrence of depression and anxiety may decrease opioid responsiveness in patients with shortness of breath.^107^

Patients in the M‐S‐Severity class and Both classes reported clinically meaningful decrements in cognitive function. These findings are consistent with studies of patients with COPD that reported negative associations between the severity of shortness of breath and cognitive impairment.^108^, ^109^ In terms of mechanisms, in the recent meta‐analysis,^110^ higher levels of hypoxemia were associated with more severe cognitive decline in patients with COPD.

### 4.4 Stress and resilience

#### 4.4.1 Common risk factors

Patients with the three worse profiles reported higher levels of cancer‐specific stress including higher levels of intrusion and hyperarousal. Across the three worse classes, 49.1%–60% of the patients had total IES‐R scores that suggest clinically meaningful PTSD symptomatology and 35.2%–40% had scores suggestive of probable PTSD. These percentages are higher than the 20.5% reported by individuals with chronic widespread pain^111^; 28.3% in individuals who were diagnosed with COVID‐19^112^; and 9.6% of patients with breast cancer.^113^

One possible explanation for this finding is that alterations in the hypothalamic–pituitary–adrenal (HPA) axis may increase the levels of pro‐inflammatory cytokines with associated airflow limitations that may increase the severity of shortness of breath.^114^ For example, in a study of the German general population, compared to individuals without PTSD, those with PTSD were at increased risk of developing airflow limitations.^115^ Future studies need to investigate how dysregulation of neuroendocrine stress axes and increased allostatic load may influence the perceptions of shortness of breath. In addition, the occurrence of PTSD may influence a patients' cognitive‐emotional responses to shortness of breath.^116^ Given the established interrelationship among systemic inflammation, disruptions in the HPA axis, and the occurrence of PTSD,^117^ dysregulation of neurotransmitters in the limbic system may contribute to the perceptions of higher levels of distress from shortness of breath.^118^, ^119^

#### 4.4.2 Distinct risk factors

Distinct risk factors associated with membership in the worse distress and Both High classes included higher levels of global stress and higher avoidance scores. In addition, higher cumulative life stress was reported by patients in the Both High class. The positive associations between global stress and shortness of breath are consistent with studies of patients receiving supportive care,^28^ workers exposed to silica,^120^ and COVID‐19 survivors.^121^ While no studies of oncology patients were identified, these associations may be mediated by higher levels of depressive symptoms and/or anxiety.^122^ On the other hand, individual variations in the use of coping strategies or social support may moderate these associations.^123^, ^124^

Evidence suggests that early life stress contributes to blunted cortisol responses by altering the responsiveness of the HPA axis.^114^ Reduced inhibitory feedback associated with stress contributes to airway sensitization, systemic inflammation, and alterations in neurotransmitters in the limbic system that may influence more severe and distressing shortness of breath.^114^, ^115^, ^118^ While not evaluated in oncology patients, in a study of individuals with chronic pain who had low interoceptive accuracy,^125^ they reported higher levels of depressive symptoms and anxiety. Of note, these individuals had difficulty distinguishing the sensory perceptual and affective dimensions of pain and attributed pain‐related distress to emotional distress.^126^

While no between group differences in resilience scores were found, all of the classes had mean CDRS scores below the normative score for the United States population. While our a priori hypothesis was not supported, one plausible explanation for this finding is that regardless of the severity or distress of shortness of breath, resilience scores are relatively low in patients with this symptom.^33^ Likewise, in a study of patients with pulmonary disease who were receiving oxygen,^127^ half of them reported resilience scores that were lower than the Nordic general population.^128^

## 5 LIMITATIONS

Several limitations warrant consideration. Given that our sample was relatively homogenous in terms of race, ethnicity, and gender, our findings may not generalize to more diverse patients. In addition, given the relatively small sample sizes for the higher classes, our findings warrant replication. Given the heterogeneous types of cancer and chemotherapy regimens in the current sample, future studies need to assess the occurrence, severity, and distress of shortness of breath in patients with specific types of cancer, and chemotherapy regimens. In addition, it would be interesting to replicate these findings in patients with and without cancer who are receiving palliative care. While this study used a valid and reliable measure to assess the subjective experience of shortness of breath, future studies need to evaluate for correlations with objective measures (e.g., pulmonary function tests and neuroimaging). In addition, detailed information is needed on the etiology of shortness of breath and the use of strategies to manage shortness of breath (e.g., use of oxygen).

## 6 CONCLUSIONS

This study presents new evidence on a comprehensive list of risk factors that influence the severity and/or distress of shortness of breath. Clinicians can use these findings to identify patients at increased risk for more severe and distressing shortness of breath regardless of their cancer diagnosis. Additional studies are needed that evaluate targeted interventions for these two symptom dimensions. Equally important, mechanistic studies of each dimension are warranted to guide the development and testing of tailored interventions.

## AUTHOR CONTRIBUTIONS

**Joosun Shin:** Conceptualization (equal); data curation (equal); formal analysis (equal); methodology (equal); writing – original draft (lead); writing – review and editing (equal). **Marilyn Hammer:** Conceptualization (equal); data curation (equal); investigation (equal); validation (equal); writing – original draft (equal); writing – review and editing (equal). **Mary E. Cooley:** Conceptualization (equal); supervision (supporting); validation (supporting); writing – review and editing (supporting). **Bruce A. Cooper:** Conceptualization (equal); data curation (equal); formal analysis (lead); funding acquisition (supporting); investigation (equal); methodology (equal); project administration (supporting); software (lead); writing – review and editing (equal). **Steven M. Paul:** Conceptualization (equal); data curation (equal); formal analysis (equal); methodology (equal); writing – review and editing (equal). **Frances Cartwright:** Conceptualization (equal); methodology (equal); validation (equal); writing – review and editing (equal). **Kord M. Kober:** Conceptualization (equal); supervision (supporting); validation (supporting); writing – review and editing (equal). **Yvette P. Conley:** Conceptualization (equal); investigation (equal); validation (equal); writing – review and editing (equal). **Jon D. Levine:** Conceptualization (equal); methodology (equal); validation (equal); writing – review and editing (equal). **Christine Miaskowski:** Conceptualization (lead); data curation (lead); formal analysis (equal); funding acquisition (lead); investigation (equal); methodology (equal); project administration (lead); resources (lead); software (equal); supervision (lead); validation (equal); visualization (equal); writing – original draft (equal); writing – review and editing (equal).

## ETHICS STATEMENT

This study was approved by the Committee on Human Research at the University of California, San Francisco and by the Institutional Review Board at each of the study sites.

## Data Availability

Data are available from the corresponding author following the completion of a data sharing agreement with the University of California, San Francisco.
